# Hearing the Voices of Australian Thyroid Cancer Survivors: Qualitative Thematic Analysis of Semistructured Interviews Identifies Unmet Support Needs

**DOI:** 10.1089/thy.2023.0080

**Published:** 2023-12-07

**Authors:** Christine J. O'Neill, Melissa A. Carlson, Christopher W. Rowe, Elizabeth A. Fradgley, Christine Paul

**Affiliations:** ^1^ Surgical Services, John Hunter Hospital, Newcastle, New South Wales, Australia.; ^2^ School of Medicine and Public Health, University of Newcastle, Newcastle, New South Wales, Australia.; ^3^ Hunter Medical Research Institute, Newcastle, New South Wales, Australia.; ^4^ Department of Endocrinology, John Hunter Hospital, Newcastle, New South Wales, Australia.

**Keywords:** thyroid cancer, health-related quality of life, thyroidectomy, cancer survivorship

## Abstract

**Background:**

Most thyroid cancer survivors regain their physical health-related quality of life, but psychological and social deficits persist. The nature of these detriments remains poorly understood and they are insufficiently captured by survey data alone. To address this, qualitative data exploring the breadth and depth of thyroid cancer survivors' experiences and priorities for supportive care are required.

**Methods:**

Twenty semistructured interviews were undertaken with a purposive, maximum variation sample of thyroid cancer survivors. Interviews were transcribed verbatim and coded independently by two researchers. A hybrid model of inductive and realistic codebook analysis was undertaken with themes developed.

**Results:**

Patient experiences centered around three themes: (1) impact of diagnosis and treatment, (2) thyroid cancer does not happen in isolation, and (3) role of clinicians and formalized support structures. The word “cancer” had negative connotations, but for many, the reality of their experience was more positive. Despite feeling “lucky” at the relative low-risk nature of thyroid cancer, many patients reported fatigue, weight gain, and difficulty returning to usual activities; concerns that were largely dismissed or minimized by clinicians. Few were offered any support beyond their treating doctors; where patients attempted to access formalized supportive care, little was available or appropriate. Life stage and concurrent family and social stressors greatly impacted patients' ability to cope with diagnosis and treatment. Addressing thyroid cancer in isolation felt inappropriate without appreciating the broader context of their lives. Interactions with clinicians were largely positive, particularly where information was communicated as a means of empowering patients to participate in shared decision-making and where clinicians “checked in” emotionally with patients. Information about initial treatments was largely adequate but information on longer term effects and follow-up was lacking. Many patients felt that clinicians focused on physical well-being and scan results, missing opportunities to provide psychological support.

**Conclusions:**

Thyroid cancer survivors can struggle to navigate their cancer journey, particularly with regard to psychological and social functioning. There is a need to acknowledge these impacts at the time of clinical encounters, as well as develop information resources and support structures that can be individualized to optimize holistic well-being for those in need.

## Introduction

Despite good clinical outcomes, thyroid cancer survivors experience significant health-related quality of life (HRQoL) detriments.^1–4^ Although HRQoL improves over the 1st year following diagnosis,^5,6^ long-term deficits in psychological and social well-being frequently persist.^7–10^ It is critical to understand the broader implications of thyroid cancer survivorship to optimize all dimensions of patient well-being.

Studies in thyroid cancer survivorship have utilized a wide variety of generic and thyroid-cancer specific survey tools (patient-reported outcome measures [PROMs]) at various time points.^9,11–16^ There is no consensus as to which PROM is superior^12,17^ and the nuances of the thyroid cancer experience cannot be completely captured by predefined questions alone. Some PROMs focus on physical and psychological outcomes and lack questions regarding social functioning^12,13,17^ or financial hardship.^18–21^ Qualitative research ranks issues such as fear of cancer recurrence (FCR), social supports, and employment as highly important,^22–24^ and lack of knowledge, clinician distrust, and poor communication as factors that may impair HRQoL.^25–28^ Socioeconomic factors may affect access to health care, coping strategies and lead to gaps or delays in diagnosis, treatment, and survivorship care.^29–32^

These issues may drive poor HRQoL and are poorly addressed by currently available PROMs.^11^ To understand the extent and breadth of thyroid cancer survivorship, qualitative data are essential.

This study documents the free expression of patients' experiences and priorities regarding their thyroid cancer survivorship, specifically exploring issues around FCR, financial hardship, social impacts, support structures, and the relevance and adequacy of information. We hypothesize that these data will provide a framework for designing interventions to improve holistic well-being in thyroid cancer survivors.

## Methods

### Participants and setting

Telephone interviews were undertaken as part of a mixed-methods study of HRQoL in a sample of thyroid cancer survivors. PROMs (Short-Form 12; European Organisation of Research and Treatment of Cancer; City of Hope Quality of Life-Thyroid Version; Thyroid Cancer Quality of Life Survey; Patient Health Questionnaire 4; Assessment of Survivor Concerns Scale) were obtained at 0, 3, 6, and 12 months after diagnosis (financial toxicity also measured at 3 and 12 months). This broader study recruited 75 patients with newly diagnosed thyroid cancer (excluding anaplastic and incidental micropapillary thyroid cancer with no adverse features) during 2021 and 2022, from Hunter New England Local Health District (HNELHD), New South Wales, Australia. HNELHD has an area of 131,785 km^2^ with an estimated population of 920,000, spanning metropolitan, regional, and rural areas. Patients underwent surgery with high-volume thyroid surgeons who participated in the area-wide multidisciplinary team meeting.

An initial pilot of the interview script was undertaken in October and November 2020 with eight thyroid cancer survivors, 1–20 years after diagnosis (not part of the wider PROM study). The pilot interview guide was reevaluated after thematic analysis of the first eight interviews; it was largely unchanged, and hence, the original eight interviews were included for analysis. Fifteen patients from the wider PROM study were selected through purposive, maximum variation sampling regarding age, gender, tumor stage at presentation, and geography. Three patients declined due to the time commitment required. There were fewer than five patients with medullary thyroid cancer or undergoing active surveillance in the wider PROMs study: these were not invited to interviews.

The subsequent 12 interviews were undertaken in February and March 2022 with patients 6–12 months following their thyroid cancer diagnosis. The study was approved by the Hunter New England (2019/ETH13770) and University of Newcastle (H-2021-0097) Human Research Ethics Committees.

### Interview design and data collection

Participants were approached and interviews undertaken via telephone at a time and place of convenience to the patient. Two female, trained research assistants (M.C. and P.B.) with at least 1 year of full-time experience in patient-centered study interviews in mixed oncology populations undertook the interviews. They were unknown to participants and not involved in clinical care. Consent was recorded for all participants and brief field notes were taken. Patients were encouraged to express their experience in an unstructured manner with questions such as “What impact has thyroid cancer had on your life?” Where needed, follow-up prompts explored issues identified by a literature review including the following: FCR,^10,22,33^ finances,^18–21^ emotions,^10,34,35^ impacts on family and social interactions,^4,36^ professional support structures,^37^ employment and daily activities,^22,32^ and timing and adequacy of information^8,38^ (Supplementary Appendix SA1).

### Data analysis

Interviews were recorded and transcribed verbatim. Data were independently coded by two investigators (C.J.O. and M.C.) with different backgrounds (clinical and sociology). A hybrid model of inductive and realistic codebook thematic analysis was utilized as described by Braun and Clarke.^39^ An initial codebook was devised following the first eight interviews and content reviewed to ensure depth and richness of the data. Coding taxonomy was revised and developed further with the remaining 12 interviews. Codes and categories were discussed among the researchers until consensus was reached. Themes and subthemes were developed, revised, and retested against the data. Participants did not review transcriptions or provide feedback on findings.

Although the research team did not aim for thematic data saturation (as suggested by Braun and Clarke)^40^ the data set was deemed of sufficient depth and breadth with no new categories identified after 16 interviews. Data were stored and managed electronically using NVivo 11 (QSR International, Melbourne, Australia).

## Results

Twenty interviews were completed, ranging from 20 to 66 minutes of duration. Demographic, disease, and treatment characteristics of the 20 participants are summarized in Table 1. Thyroid cancer survivors' experiences are explained around the following three themes: (1) impact of diagnosis and treatment (Table 2); (2) thyroid cancer does not happen in isolation (Table 3); and (3) role of clinicians and formalized support structures (Table 4).

**Table 1.** Demographics and Disease Characteristics of Interviewees, *n* = 20

Age at time of diagnosis, years (median, range)	54 (14–77)
Gender (women, %)	13 (65)
Insurance status (private, %)	13 (65)
Geography	
Metropolitan	11 (55)
Regional	9 (45)
Educational status	
High school or equivalent	2 (10)
Vocational training	9 (45)
University degree	9 (45)
Smoking status	
Never	17 (85)
Current or ceased <2 years ago	0
Ceased >2 years ago	3 (15)
Cancer type	
Papillary thyroid cancer	15 (75)
Follicular thyroid cancer	2 (10)
Hurthle cell cancer	2 (10)
Insular cancer	1 (5)
Extent of surgery	
Total thyroidectomy^a^	9 (45)
Hemithyroidectomy	9 (45)
2 Stage total thyroidectomy^a^	2 (10)
Lateral neck dissection (in addition to total thyroidectomy)	1 (5)
ATA risk^b^	
Low	12 (60)
Intermediate	5 (25)
High	3 (15)
AJCC TNM Stage (8th edition)^d^	
Stage 1	16 (80)
Stage 2	2 (10)
Stage 3	0
Stage 4^c^	2 (10)

^a^ All of these patients also had radioactive iodine ablation.

^b^ ATA: 2015 American Thyroid Association Management Guidelines for Adult Patients with Thyroid Nodules and Differentiated Thyroid Cancer.^72^

^c^ Both patients were being treated with lenvatinib at the time of interview.

^d^ AJCC TNM: American Joint Committee on Cancer Tumor Node Metastases Cancer Staging Handbook, 8th Edition.^73^

**Table 2.** Impact of Diagnosis and Treatment: Subthemes and Exemplar Quotes

**“Cancer”: difference between prior associations and reality** Patients expressed shock at the time of diagnosis due to their prior associations with the word “cancer.” The reality of their treatment and prognosis differed from these associations with many feeling *lucky* at the low-risk nature of their disease but also reporting that some clinicians lacked empathy.
*Cancer, that's a scary word. (F,56–70,low)* *It was a shock at first to be diagnosed, but then the treatment and everything followed that quickly, it was over and done with all very suddenly. And then the prognosis, or whatever you refer to it as, from then seemed fine, everything was captured and there should be no ill effects. (M,56–70,low)* *My experience of cancer is this could be killing me and I wouldn't even know. So the I wish, after the initial diagnosis, someone had explained to me how easy it was going to be, and how it wasn't mega-life-threatening. Because it would have been a much more pleasant two weeks after the diagnosis. (F,40–55,low)* *They (the doctors) just dismissed it and I felt like I was being a drama queen because I had cancer. A little bit more empathy would have been a bit better and eased me a bit more. (F, <40,intermediate)*
**Psychological and emotional impacts as a “cancer survivor”** While many patients initially denied many impacts of their diagnosis. With further probing most admitted to some fear of cancer recurrence, change in life perspective and need for at least a basic level of emotional support during follow-up.
Initial minimization of impacts *I'm going to be a very boring person. The only impact I've had is that I take tablets every day. (F,40–55,low)* Fear of cancer recurrence *It is in the forefront of your mind sometimes, but it doesn't control or consume me by any means. (F, <40,low)* *I'm just a little bit anxious about what could happen in the future, but that's something that's out of our control anyway. (M,56–70,low)* Change in life perspective *I never thought so much about death until this happened. So it reminds you that you're not here forever. (F,40–55,low)* *Sometimes you hear someone dying of cancer and you think you never know what is around the corner. So, you have got to enjoy today. That is my motto I suppose. Other things I have been putting off and now I'm not. (M,56–70,low)* *I feel like I was one of the lucky ones that it was caught so quickly. I definitely don't take life for granted, that's for sure. (F, <40,intermediate)* Emotional response to clinical follow-up *Those appointments can be worrying, bring up a little niggle perhaps in the back of your mind, but it's reassuring to be checking in and knowing things are still on track. (M,56–70,low)* *The check-ups provide a sense of security, but if I walked away and had no more check-ups that would be better. (F,40–55,low))* *It's just about your physical health. What's your problems and why. No-one really asks you if you're OK or how are you feeling? (F, <40,intermediate)*
**Disparities in access to care and impact of COVID-19** The time between diagnosis and surgery differed for many patients with some feeling “rushed through,” while others felt additional emotional distress due to delays in appointments and surgery, and remote care exacerbated by the COVID-19 pandemic.
Access to care *It threw me back a little bit that it happened so quickly because it makes it a bit scary. Why are they doing it so quick? (F,56–70,low)* *Just wait and have it done when your name comes up. I feel like I had to push for things to happen sooner. (F, <40,low)* Impact of COVID-19 with delays in care, increased isolation, and telehealth consultations *Everything was remote, not actually seeing someone felt a bit strange, especially for a cancer thing. (F,40–55,low)* *Not being able to see someone, you don't get to speak at length, you don't necessarily gather your thoughts quickly. So questions like alternative ways to look for cancer or manage risk, I put off. (F,40–55,low)*
**Discordance between patient and clinician perceptions of magnitude of effects** Patients reported ongoing concerns regarding weight gain, voice change, thyroxine adjustment, cognitive effects, and fertility that were unacknowledged, minimized, or dismissed by their treating clinicians.
Differing responses to diagnosis and recovery *They need to get rid of their assumptions and just be straight to the point that it's different for everybody, you might be right in a month, you might not be right in two years, it's very much a personal journey. (F, <40,intermediate)* Weight gain *I struggled with weight before and had actually lost ten kilos. I had my surgery and then I gained ten kilos plus after. I found that quite depressing because I know when I gain weight and I don't like it. (F,56–70,low)* Voice *My voice came back after a month. I wasn't myself until my voice came back. (F,40–55,low)* *In my younger days I was a singer in a band. It's really hard when I get in the car, put the music on and I can't sing along any more. (M,56–70,high)* Cognition *I have massive brain fog. There's so much to do but I just can't get myself organised. (F, <40,intermediate)* Thyroxine titration *It was just frustrating that you were telling them, and they were just like, no, we're the professional, we know what we're talking about. It's like, no, it's my body, I know what I'm talking about. (F, <40,intermediate)* *We are all on completely different doses and live such different lifestyles and all have different symptoms. (F, <40,low)* Fertility *It has been over six months now trying for a baby and nothing's happening. With my endocrinologist, I went and got blood tests done to see when I was ovulating and everything like that. But I did the blood tests and he said, you are ovulating, but then that was that. So, I'm in limbo now. I'm like, well, what's the next step? (F, <40,intermediate)*

To contextualize each quote, it is identified in brackets by: (gender, age range, and ATA^a^ risk).

^a^ ATA: 2015 American Thyroid Association Management Guidelines for Adult Patients with Thyroid Nodules and Differentiated Thyroid Cancer.^72^

**Table 3.** Thyroid Cancer Does Not Happen in Isolation: Subthemes and Exemplar Quotes

**Family, social, and financial impacts** Patients' experience of their diagnosis, treatment, and recovery was impacted by their stage of life, family situation, workforce participation, and financial situation. Coping strategies and supportive care needs differed depending on the context of each individual patient.
Concurrent stressors *I was diagnosed while studying and living away from home. It was too much. (F, <40,low)* *I was trying to fix up my house as well as mum dying and getting over my cancer operation. (M,56–70,low)* Those with children *To try and explain to a two-year old what's going on with mummy, that was quite hard to do. (F, <40,intermediate)* Employment *I'd go for interview, mention that I have cancer and people would just stop the interview, put a big X next to your name. (F, <40,low)* *I just thought, the wound is healed so I'll be right to go back to work. It wasn't as simple as that. (M,56–70,low)* Finances *You don't qualify for the disability and sickness payment unless you have cancer for two years or you're terminal. (F, <40,intermediate)* *Financially it hasn't cost us anything, it's only the cost of the fuel. (M, >75,high)*
**Coping strategies and support structures** Many patients drew on prior life experiences and existing networks to gain support or to cope with their diagnosis and treatment. Formal support structures and professional support were not offered, available, appropriate, or timely.
Coping strategies—positive outlook, avoidance, and distraction *I take things as they come rather than get bogged down with worry about what could happen. (M,56–70,low)* *There was something nasty there but it's been gobbled up, spat out and we've moved on. (M,56–70,low)* *We don't ask for timeframes anymore. We just want to know what the next step is and why we're doing this. (M,56–70,high)* *I had a lot of other stuff going on at the time too, so I was a bit distracted. (F,40–55,low)* Preexisting support structures *In times like that you just have call in favours and bring your community around you and get help. (F,40–55,low)* *I told my team at work. And they were very supportive and just told me to do whatever you need. (f,40–55,low)* *I felt like I did a lot of upfront happy faces in front of family and friends to reassure them but then fell apart in front of my husband. Yes, so he was probably the biggest support that I had. (F, <40,low)*

To contextualize each quote it is identified in brackets by: (gender, age range, and ATA^a^ risk).

^a^ ATA: 2015 American Thyroid Association Management Guidelines for Adult Patients with Thyroid Nodules and Differentiated Thyroid Cancer.^72^

**Table 4.** Role of Clinicians and Formalized Support Structures: Subthemes and Exemplar Quotes

**Clinical reassurance and information** Many patients derived significant reassurance and support from their interactions with clinicians. Those who expressed trust in their clinicians and felt informed reported less psychosocial concerns. Information around initial treatment was adequate but there were notable gaps in the content and timing of information beyond the initial treatment phase.
Clinician reassurance *I hope (the endocrinologist) stays my specialist because I really feel that (they) care and want to inform me of everything, which I really value. (F, <40,intermediate)* *A critical part in my journey was (the surgeon) making me feel so at ease with it. (F,40–55,low)* *The health professionals, we've been seeing them on a fairly frequent basis, that's been all the support we've needed. (M,56–70,high)* Adequacy of information (written and verbal) *It's all (the surgery) explained in the coloured, nice paper brochure they hand you when you see your surgeon. Since then, I haven't learnt anymore, no one's been able to elaborate any further. Maybe there is no more information. (M, >70,high)* *Looking back in hindsight there's probably times when I would have liked to have known more about what I should be doing as far as monitoring drugs, thyroxine levels and scanning for cancer and whatever I should take, the radioactive iodine or not, that type of thing. (M,56–70,low)* *I feel like knowledge is power and I'm all about being proactive. (F, <40,intermediate)* *They (the surgeon) had excellent skills in making me feel comfortable and providing me with all the information I needed to make a decision about which course of treatment I would take. (F,40–55,low)* Timing of information *They give you pamphlets and say go home and read that. That is fine, but I suppose when you are first told you have cancer you step back three steps. So, when they say have you got any questions and at that time you sort of go no, what do you think and just virtually run off what they are trying to tell you. It is probably three or four days later when you get home you say should I ask this or should I ask that. (M,56–70,low)* Information gaps and individualization of information *Are there things that I need to be aware of? Information about living with thyroid replacement medication and what's going to happen to your body; I've put on weight, people get a lot of fatigue, how to manage the heat, what to eat. Is that my thyroid or is that just me? (M,56–70,low)* *I didn't feel like I got a lot of information, I had to do a lot of research myself online to find out what was going on. And even with treatment, and getting support, I had to go online and social media to try and find people my own age that had similar experiences with this, to see what they find works, what they find doesn't work. (F, <40,intermediate)*
Formal support structures
Patients were asked what support structures they would have valued and if they had tried to access them: *I wasn't even referred to the Cancer Council, or any support groups, so it was completely up to me to find support for myself. (F, <40,intermediate)* *I've had that many different people, you really need a central coordinator somewhere that you can contact, that knows the answers to a lot of the questions. (M,56–70,low)* *I think I should go see a psychologist. I tried about six months ago, but the waiting lists… a lot of people weren't taking on new people so I just gave up. (F, <40,low)* *I know there's support out there, but no one's actually come forth and offered any. But I haven't been seeking it either - you know, you get the little leaflets at the hospital. If you want support I know it's all out there. (M, >70,high)* Regarding timing of supports *Months later, it's forgotten about and that's when it really starts to sink in. (M,56–70,low)*

To contextualize each quote it is identified in brackets by: (gender, age range, and ATA^a^ risk).

^a^ ATA: 2015 American Thyroid Association Management Guidelines for Adult Patients with Thyroid Nodules and Differentiated Thyroid Cancer.^72^

### Impact of diagnosis and treatment

#### Differences between reality and prior associations with the term “cancer”

Most patients expressed shock at the time of diagnosis, associated with the term “cancer.” Some patients reported receiving their diagnosis with little additional explanation or information, and experienced significant distress as a result. However, the subsequent reality of diagnosis, treatment, and prognosis was more positive than their prior associations with cancer. Many patients felt “lucky” about the relative low-risk nature of thyroid cancer or of its early detection: “Out of all the cancers you could get, you win the cancer raffle!” (F,40–55,low).

#### Psychological and emotional impacts as a “cancer survivor”

When asked initially, most patients minimized any physical, psychological, or social impacts of their diagnosis. However, with probing, most admitted FCR but reported minimal impact on day-to-day life. Many described alterations in life perspective and distress about changes in their locus of control. These “fears” related to prior associations and personal experience with cancer-related death, as well as a reminder of the unpredictability of the future. While causing temporary anxiety, most found that follow-up provided reassurance and a sense of “control” over their disease. For some, however, follow-up was anxiety provoking and provided little reassurance: they would have preferred to walk away. There was also a sense that follow-up was more about scans, blood tests, and physical health and that opportunities to improve psychological well-being were missed or undervalued.

#### Disparities in access to care and impact of COVID-19

Uninsured, regional, and rural patients reported delays in accessing specialist care. This compounded the distress associated with their cancer diagnosis and many felt the need to be proactive, advocating for themselves to obtain and expedite treatment. On the contrary, some (mostly privately insured) patients gained rapid access to specialist appointments and surgery leading to a feeling of being rushed through the process without time to fully digest their diagnosis and treatment options. The impact of the COVID-19 pandemic and government restrictions was felt with delays in care, increased isolation, and telehealth consultations: “I couldn't go in and see [the surgeon], so that was hard” (F,40–55,low). Patients expressed increased stress due to separation from family and social supports while in hospital and felt telehealth consultations failed to provide as in-depth information or reassurance as a face-to-face consultation.

#### Discordance between patient and clinician perceptions of magnitude of physical, cognitive, and emotional effects

Postoperative effects including voice change, swallowing difficulty, scarring, and calcium deficiency frustrated some patients for many months postoperatively. The time frame of recovery was significantly longer than anticipated. These lingering symptoms delayed their return to social functioning and usual activities. Two patients had high-risk thyroid cancer and were on lenvatinib, one received adjuvant external-beam radiation and the other required recurrent laryngeal nerve sacrifice at surgery. In contrast to low-risk patients, these patients received ongoing treatment and support for their persisting physical symptoms.

Many patients felt that clinicians dismissed or diminished their concerns. “It's an everyday thing for them, it's not a biggie, whereas to the average person like myself it is” (M,56–70,low). Information about managing thyroid function (with or without thyroxine) was reported as inadequate, and concerns regarding weight gain, changes in motivation, fatigue, concentration, and activity levels were dismissed or minimized by clinicians “it would have been nicer for them to just acknowledge that it was a concern for me rather than brushing it off” (F, <40,intermediate).

Patients expressed frustration that what seemed small (or expected) effects to clinicians were highly significant within the context of their lives and prevented their ability to return to work or study. These effects were not anticipated by most, nor mentioned as part of their recovery plan. Little support was available, advice was lacking, and the significant mental strain experienced by patients was unacknowledged, dismissed, or minimized by clinicians. Examples included weight gain for patients who had previously struggled with weight; memory, concentration, and fatigue issues for students or those in high-pressure professions; and voice change for singers and public speakers. In addition, women reported that information regarding fertility focused purely on physical impacts and deferring pregnancy after radioactive iodine. This failed to adequately inform or reassure women regarding future pregnancy.

### Thyroid cancer does not happen in isolation

#### Family, social, and financial impacts

Concurrent health issues or social stressors were frequent, and compounded patients' ability to cope with their thyroid cancer diagnosis. Patients with young children and those in the workforce expressed more family and social impacts. Parents struggled to explain their diagnosis to their children and with separation from children following radioactive iodine. Most found their work environments supportive and benefited from flexible work arrangements due to the coincident timing with the COVID-19 pandemic. Others found difficulty reentering the workforce due to prejudice associated with cancer and concerns regarding their ability. Prolonged physical effects, fatigue, and lack of motivation affected reengagement with employment and usual activities.

Patients were questioned specifically about the financial cost of treatment and recovery. Most denied significant financial concerns despite admitting out-of-pocket expenses. Financial impacts were greater for those who had to travel for treatment or for those unable to work.

#### Coping strategies and support structures

Many patients described a “positive outlook” (F,56–70,low) and distraction as coping strategies: “I just tend to get on with things, I don't let things get me down” (M,56–70,high). Support was gained from family and friends as well as employers, particularly around the time of diagnosis and surgery. Some patients used their wider family and social networks for support, while others relied more heavily on their partner, trying to “normalize” the experience for their wider networks.

### Role of clinicians and formalized support structures

#### Clinician reassurance and information

Many participants expressed significant trust in, and rapport with, treating clinicians: “they are the ones who have the 6, 8 years and probably more of study, so you have to leave a lot of it in those people's hands” (M,56–70,low); and “you feel like you're more at home and you can trust them a bit more because they generally do care” (M,56–70,high). Patients expressed relief after their first surgical appointment where they began to understand the “low-risk” nature of their cancer diagnosis. Many also commented on the communication skills of their treating clinicians, which empowered them with confidence to participate in shared decision-making regarding their treatment choices. Patients appreciated clinicians who “checked in” emotionally with them during follow-up consultations and provided time to voice any concerns.

Most were satisfied with verbal and written information regarding side effects and risks of treatments (surgery, radioactive iodine, and lenvatinib), but felt there were notable gaps regarding the risk of recurrence, the rationale and benefits of radioactive iodine, long-term effects of treatment, and the process of follow-up. Some felt that information was not timely or was unsuitable for their individual circumstances. This led to feelings of disempowerment and with patients sourcing information from friends, family, and the internet. Specific information gaps included the impacts of thyroid medication on diet, weight gain, activity, and fertility; the same concerns that patients felt were significant to them but minimized by clinicians. The timing of information at diagnosis was adequate for most, but later on, when ongoing impacts of diagnosis and treatment were felt, little information was available and contact with clinicians was infrequent.

#### Formalized support structures

Most patients were not offered support services, and some were discouraged from making contact. When prompted, most admitted that they would have declined additional support if offered at the time of diagnosis but that it was most required months later. Some sought support online or attempted to access psychology or cancer coordinator services. Attempts to access supportive care were hampered by a lack of accessible pathways, leaving many without timely and appropriate assistance.

## Discussion

These data describe the breadth of thyroid cancer survivorship, giving patients a “voice” to explain their experiences, in their own words. Collectively, it highlights the need to individualize care, acknowledge the wider impact of thyroid cancer on patients' lives, and provide informal and formal support. Patient experiences varied considerably. For many, physical recovery was prolonged and ongoing psychological and emotional concerns prevented full reengagement with work and social activities. Despite many feeling “lucky” at the low-risk nature of their diagnosis, some simultaneously felt dismissed for having a “good cancer” and that concerns regarding their recovery were minimized by clinicians.

### Patient needs at the time of diagnosis and treatment

In line with prior research, patients' described strong emotional responses to the term “cancer.”^41,42^ Those who developed trust in their health care providers, received emotional support, and were empowered with information to participate in treatment decisions reported less psychosocial impacts.^43,44^ Patients expressed either a strong desire to avoid thyroid hormone replacement or to minimize cancer recurrence. As in prior studies,^45,46^ those who felt that these values were explored when considering treatment, expressed satisfaction with their treatment choices, regardless of the extent of surgery. These findings are critically important in the era of shared decision-making, where the information needs and longer term HRQoL priorities must be considered when deciding on the extent of surgery for low-risk thyroid cancer.^26,47,48^

### Patient needs beyond the initial treatment period

While sentiments regarding a “good cancer”^28,49^ and being “lucky”^50^ have been described in previous studies, the discordance between clinicians and patients regarding the magnitude of impacts on holistic well-being was prominent in this study. Persisting fatigue, cognitive deficits, voice change, and weight gain are common themes in other thyroid cancer survivorship studies.^3,7,27,36,51,52^ It is of concern that, despite strong evidence that these symptoms drive HRQoL detriments, opportunities for these effects to be acknowledged and addressed by clinicians are being missed.

While patients drew significant support from existing family and social networks, there were significant information and supportive care gaps. In line with prior research, patients reported a change in life perspective following their diagnosis^53^ but by the time they were ready to process their diagnosis and resume normal life, their appointments were infrequent and they lacked pathways to access information and supportive care. For thyroid cancer survivors, studies have shown improvement in overall well-being with nurse navigator coordinated care,^37^ and with psychological^54^ and mindfulness-based^55^ interventions. These multidisciplinary support structures and care coordinators are often available for other cancer patients,^56,57^ but not thyroid cancer survivors. Patients also lacked information on persisting symptoms and follow-up care as well as pathways to source reliable information for issues specific to their clinical context or personal situation.

This finding is consistent with studies in thyroid^16,38,58,59^ and other cancer types,^60–63^ and highlights the need to continually incorporate individuals' preferences, information needs, and values into all aspects of patient-centered care.

Prior research suggests FCR as a driver of poor HRQoL in thyroid cancer survivors^9,10,22,33,64^ and that follow-up can increase this anxiety.^23^ In this study, while the majority found clinical follow-up reassuring, and that it decreased their FCR, some would have preferred less follow-up. There is little evidence that long-term follow-up in low-risk thyroid cancer provides disease-survival benefit or is cost-effective.^65,66^ Thus, clinician follow-up should provide an opportunity for psychosocial support including FCR screening, normalization, and education. Where FCR is moderate or severe, patients should be referred for formal psychosocial support.^67–70^

The major strength of this study is the literature-based design that is aligned with a separate PROM study. The data here give patients a voice beyond survey data alone. The themes presented would not have received sufficient emphasis if presented alongside survey data. Our findings should be interpreted within study limitations. Despite patient selection to include demographic and geographic diversity, we cannot be certain that our sample is truly representative of the wider thyroid cancer population. In particular, the views of those with medullary thyroid cancer or undergoing active surveillance were not captured. We accept that demographic concordance can affect patient communication, participation, and satisfaction and that this may influence responses.^71^

Subjective experiences can shape interpretation of the data. While this was limited by research team members having diverse views and backgrounds (clinical, sociology, behavioral science), and engaging in reflexive discussion throughout analysis and theme development, it is possible that some unique issues were not apparent.

## Conclusions

Thyroid cancer survivors experience broader impacts to their lives than is widely acknowledged. Many experience prolonged recovery that delays return to their prior level of functioning. Clinicians should be alert to the need for individualized support beyond the initial treatment phase and that follow-up should address patients' psychosocial functioning and not just clinical findings and test results. Appropriate and accessible information and support services are needed, both at diagnosis and subsequently.

## Supplementary Material

Supplemental data

## Supplementary Material

Supplementary Appendix SA1
