# Supplementary material for: Hearing the Voices of Australian Thyroid Cancer Survivors: Qualitative Thematic Analysis of Semistructured Interviews Identifies Unmet Support Needs
Source: Thyroid. 2023 Dec 7;33(12):1455–64. doi: 10.1089/thy.2023.0080 (PMC10734898; doi:10.1089/thy.2023.0080)
Supplement: Supplemental data [file Suppl_AppendixSA1.docx]

**Appendix 1: Interview guide**

The interview guide design was guided by a review of the literature and our own prior research in Australian thyroid cancer survivors.(5)

***Introduction***

*‘We want to talk through some aspects of what it means to be a thyroid cancer survivor. You have done the surveys online and will have noticed that many of the questions are about how the diagnosis of thyroid cancer has affected your quality of life. We want to discuss some of these issues in more depth today and to focus on any support you have had. Before we start more specific questions it would be good to talk to you about the impact that thyroid cancer has had on your life. Could you tell me a bit about that?’*

*Prompts:*

- *How has it affected your family?*
- *How has it affected your social life or interactions with others?*
- *How has it impacted your finances?*
- *How has it affected you emotionally?* How has it impacted on your physical health?

1. *‘You will have noticed that the surveys were designed to capture areas where people might have worries and concerns. One of those concerns can be about whether the cancer will come back. How much do you think about whether your cancer might come back?*

*Prompts*

- *When it does come to mind is it a big worry, or more of a passing thought?*
- *When you think about it coming back how does that affect your daily life or your mood?*

*Would you be able to tell me some of the worries that you have if the cancer comes back?*

- *Has your view on life changed since your cancer diagnosis?*

1. *At any time since your diagnosis has any health professionals discussed with you the specific risk of your cancer coming back?*
2. Which health professional/s spoke to you about this?
3. Who started the conversation?
4. What information were you given?
5. Did you find that this information reduced your concerns, or did it increase your anxiety about your cancer coming back?
6. Was there anything about the way the information was delivered that made you more anxious/ helped make you less anxious

If the ppt was NOT given any information about the risk of recurrence:

1. Would you have liked to have been given information on the risk of recurrence? Why/Why not?
2. Who would you feel comfortable hearing this information from? (surgeon, endocrinologist, GP, etc.)
3. When would it have been most helpful for you to hear about the risk of recurrence?
4. *‘Have you had any follow-up appointments for your thyroid cancer yet?’ How do these appointments affect your level of concern about the cancer coming back?’*

*Prompts:*

- *How do follow-up appointments affect your emotional well-being?*
- *If there was an option of changing how often you have follow-up appointments, would you find that less stressful/more reassuring (tailor to prior response)*
- *Is it the tests involved that are helpful/unhelpful or the clinician appointments*
- *Do you have concerns about your health?*
- *Do you have concerns about a second cancer?*

1. *How do you think your cancer diagnosis has affected your family?*

*Prompts:*

*- Does the thyroid cancer raise concerns for you about your family’s health?*

*- How much opportunity is there for you to discuss these concerns with family members?*

1. *Are there any other concerns that you have about your cancer coming back or your health that we have not covered so far*

*Now I would like to discuss what supports you had around the time of your thyroid cancer diagnosis and since this time. The support might have come from a variety of sources e.g. family, friends, professionals, or volunteers.*

1. *Can you tell me about how you have been supported during the time around diagnosis up until now?*

*Prompts*

- *(If focus on one type) How about other types of support?*
- *Who supported you most around the time of your thyroid cancer diagnosis?*
  - *What impact did your diagnosis have on them?*
- *Was there a point or a period of time when you needed support and didn’t get it? – tell me about that*

1. *At any point since your diagnosis were you offered professional support such as referral to a counsellor or social worker?*
   1. *If so tell me about what kind of support was offered and how that happened*
   2. *If not – do you think this might have been useful?*
   3. *What do you think might have been beneficial?*
2. *Thinking back to the time around when you were diagnosed, how well informed did you feel about your diagnosis and treatment options?*
   1. *What information did you receive – verbal/written and from whom did you receive it*
   2. *Was there anything about the way the information was given to you that made it easier or harder for you to take it all in and consider your options?*
3. *Did you feel the need to seek out additional support and if so from where?*
   1. *Was this support easy to find?*
   2. *Was this support helpful*

## **Final open-ended question of what was missing:**

‘So that is all the questions we have for you today. Is there anything else related to having a diagnosis of thyroid cancer that you would like to mention?
